# Low condom use at the last sexual intercourse among university students in sub-Saharan Africa: Evidence from a systematic review and meta-analysis

**DOI:** 10.1371/journal.pone.0272692

**Published:** 2022-08-10

**Authors:** Jonathan Izudi, Gerald Okello, Daniel Semakula, Francis Bajunirwe

**Affiliations:** 1 Faculty of Medicine, Department of Community Health, Mbarara University of Science and Technology, Mbarara, Uganda; 2 Department of Reproductive Health, Ministry of Health, Kampala, Uganda; 3 African Centre for Systematic Reviews and Knowledge Translation, Makerere University College of Health Sciences, Kampala, Uganda; The Technical University of Kenya, KENYA

## Abstract

**Background:**

There is inconsistent data about condom use at the last sexual intercourse (LSI) among university students in sub-Saharan Africa (SSA) and its association with sex, age, and condom negotiation efficacy. The primary objective of this study was to summarize the proportion of condom use at the LSI among university students in SSA. The secondary objective was to determine the association between condom use at the LSI with sex, age, and condom negotiation efficacy among university students in SSA.

**Methods:**

In this systematic review and meta-analysis, two reviewers independently searched electronic databases and grey literature for eligible studies published until July 30, 2020, extracted data, and assessed the risk of bias in the included studies. We used the Dersimonian-Liard random-effects model to pool the proportion of condom use at the LSI and the association between condom use at the LSI with sex, age, and condom negotiation efficacy, reported using risk ratio (RR). We assessed publication bias using funnel plot and Egger’s test, and explored sources of heterogeneity using sub-group and meta-regression analyses.

**Results:**

We meta-analyzed 44 studies with a combined sample size of 27,948 participants.Of 14,778 sexually active participants, 8,744 (pooled proportion, 52.9%; 95% CI, 45.0–60.7; 95% prediction interval, 2.8–98.9; I-squared = 99.0%, p< 0.0001) reported condom use at the LSI and the proportion of condom use at the LSI remained stagnant between 2000 and 2019 (*p* = 0.512). Condom use at the LSI was not associated with being a female compared to a male (pooled RR, 1.08; 95% CI, 0.68–1.71), being of a younger age (≤24 years old) compared to older age (25 years and more) (pooled RR, 1.16; 95% CI, 0-85-1.57), and having a higher condom negotiation efficacy compared to a lower condom negotiation efficacy (pooled RR, 1.54; 95% CI, 0-81-2.94).

**Conclusions:**

We found a low and heterogenous use of a condom at the LSI among university students in SSA which was not associated with sex, age, or condom negotiation efficacy. Accordingly, context-relevant interventions are needed to improve condom use at the LSI among university students in SSA.

## Introduction

In 2019, an estimated 3,400,000 young people (15–24 years old) were living with human immunodeficiency virus (HIV) globally, and two out of every seven new HIV infections were among young people [1]. Every day in 2020, global estimates indicated that 4,000 children and adults were newly infected with HIV and 60% of those infections were in sub-Saharan Africa (SSA) while 90% were among people aged ≥15 years old [2]. Young people comprised at least one-third of the daily new HIV infections among persons aged ≥15 years in 2020 [2].

In SSA, HIV remains the leading cause of morbidity and mortality among young people, and the predominant mode of transmission is condomless sexual intercourse [3]. The notable risk factors for HIV acquisition in young people include early age at sexual debut [3], multiple sexual partnerships [3], misconceptions about condom use which is worsened by negative attitudes [4], and sexual relationships with older men (age-disparate relationships) who are more likely to be HIV infected [4]. Recent data show that age-disparate relationships are associated with condomless sexual intercourse and HIV acquisition among adolescent girls and young women [5]. For that reason, risk reduction measures like abstinence from sexual intercourse, delayed sexual initiation, reduced number of sexual partners, and increased access to and use of condoms is important in preventing young people from acquiring sexually transmitted infections (STIs) as well as HIV and unwanted pregnancies [6].

University students comprise a large proportion of young and sexually active people who are at a higher risk of HIV acquisition [7]. The university environment creates a setting for high-risk sexual behaviors especially condomless sexual intercourse and multiple sexual partnerships [8].

Studies conducted in SSA have reported that a substantial proportion of sexually active university students had not used a condom at their last sexual intercourse (LSI) [7,9,10], placing them at a higher risk of acquisition of STIs including HIV. Studies have also reported that being in a steady sexual relationship [10], having a low perceived risk of HIV acquisition or indulging in unplanned sexual intercourse [9], perceiving that condoms are used solely during fertile days [10], and perceiving that condoms decrease sexual pleasure [9,10] are associated with increased likelihood of condomless sexual intercourse among university students.

Currently, there is a lack of aggregated data on condom use at the LSI among university students in SSA including the associated factors. Also, existing studies on the African continent have shown wide differences in condom use among university students, ranging from below 15% in some countries [11–14] to nearly 50% in others [15,16] and more than 75% in other countries, although from fewer studies [17,18]. In addition, the studies have shown inconsistent findings regarding the association between sex, age, and condom negotiation efficacy with condom use at the LSI [19–21]. For instance, compared to males, some studies [22,23] have reported that females have a decreased likelihood of condom use at the LSI while other studies [24,25] have reported an increased likelihood of condom use at the LSI. Based on these inconsistencies, we conducted a systematic review and meta-analysis to primarily summarize the prevalence of condom use at the LSI among sexually active university students in SSA. As a secondary objective, we determined the association between younger age (≤24 years), being a female, and having a higher condom negotiation efficacy with condom use at the LSI. This evidence will justify and inform the strategic design of condom promotion campaign programs among university students in SSA, thereby contributing to reducing HIV morbidity and mortality.

## Materials and methods

### Study design and registration

We adhered to the elements of the Preferred Reporting Items for Systematic Reviews and Meta-analysis (PRISMA) [26] (S1 Table) and Meta-analysis of Observational Studies in Epidemiology [27]. We registered the study protocol with PROSPERO and the assigned registration number is CRD42020196868 [28].

### Inclusion and exclusion criteria for studies

Our eligibility criteria included: 1) Type of participants: studies should have enrolled university students, either undergraduate or postgraduate or both; 2) Exposure of interest: being a female, younger age (≤24 years), and having a high condom negotiation efficacy; 3) Comparison group: being a male, older age (25 years and more), and having a low condom negotiation efficacy; 4) Time: all studies published until July 30, 2020; 5) Study designs: all observational studies, namely cross-sectional, case-control, and cohort; 6) Study setting: both private and public universities in SSA.

We excluded studies with the following characteristics: high risk of bias, not reported in English language, inaccessible full-text articles despite contacting the corresponding author, unclear or incorrect reporting of the primary outcome, primary outcome reported for none sexually active participants, the association between exposure and outcome reported using unadjusted odds ratio (OR), unadjusted risk ratio (RR) and unadjusted hazard ratio (HR), conducted outside SSA, and studies that combined data from a university in SSA with data from another university outside of SSA. We also excluded qualitative studies and conference presentations or abstract papers.

### Study outcomes and variables

The primary outcome was condom use at the LSI measured as the number of participants with one or more sexual partner(s) in the past 12 months (sexually active participants) who had used a condom at the LSI, expressed as a percentage. The secondary outcome was the association between being a female, being of a younger age, and having a high condom negotiation efficacy and condom use at the LSI. For the secondary outcome, we considered only the adjusted estimates for OR, RR, and HR. The two primary outcomes, “*always used a condom”* and “*consistently used condom”*, were considered synonymous with condom use at the LSI. We defined condom negotiation efficacy as the perceived capability to use condoms consistently, including how difficult a person considers it to realize the desired healthy behavior [4].

### Search strategy and process

Two reviewers (JI and DS) developed a search strategy using key concepts in the research question and for each key concept, a Medical Subject Heading (MeSH) term was developed and thereafter combined with Boolean operators: “AND”, “OR”, and “NOT”. The final search strategy used in PubMed/Medline was as follows: (“University student” OR “University students”) AND ("Condom use at last sex" OR "Condom use at last sexual intercourse" OR "Condom use at latest occasion" OR "Condom use at recent sex" OR "consistent condom use" OR "Consistent use of condom" OR "Inconsistent use of condom" OR "Inconsistent condom use" OR "Always use condom"). An example of the full search strategy in PubMed is shown in the supporting information (S1 File). Two reviewers (JI and GO) independently searched MEDLINE/PubMed, EMBASE, Web of Science, GoogleScholar, and Google iteratively for eligible studies between July 20, 2020, and August 30, 2020. The reviewers also hand-searched the reference list of eligible studies to identify other studies which were not identified by the search strategy.

In addition, grey literature like dissertations and reports were searched through LILACS and OpenGrey. We summarized and presented the results of the search strategy in a PRISMA flow chart.

### Screening of studies and data abstraction

All the identified citations were exported into *EndNote*, a bibliographic reference management software, and duplicated citations were excluded. Two reviewers (JI and GO) screened the remaining studies for eligibility using the titles and abstracts and the ineligible citations were excluded. The reviewers retrieved and read the full-text articles of eligible studies and abstracted the following data items using a standardized Microsoft Excel sheet: first author’s last name, publication year, study design, sample size, study setting, country, number of sexually active participants, number of sexually active participants that had used a condom at the LSI, and the adjusted measure of effect (OR, RR, or HR) for each of the exposure variables. In studies where the adjusted measure of effect was reported for the comparison group or no use of a condom at the LSI, we computed the reciprocal of that adjusted measure of effect to obtain that for the exposed group and condom use at the LSI, respectively. For each country, we retrieved the 2019 human development index (HDI), the most recent measure of each country’s development concerning healthcare, education, and life expectancy [29]. The measure categorizes countries into low, medium, and high HDI.

### Consensus in data abstraction and risk of bias assessment

We resolved disagreements in data abstraction through consensus with a third (DS) or fourth (FB) reviewer. To assess consistency in the screening of studies and data extraction between the two reviewers (JI and GO), we computed Kappa statistics to ascertain the degree of agreement. This is important because coding behavior changes between and within individuals over time[30].

We assessed the risk of bias in the included studies using a 9-item quality assessment checklist for prevalence studies that had a total of nine scores [31]. This tool is widely used in systematic reviews to assess the risk of bias in cross-sectional studies because it is easy to use, has a high inter-rater agreement, and a nearly perfect agreement between the individual items. The tool measures the risk of bias in each study on a binary scale (yes or no) and the nine items included whether 1) the study’s target population was a close representation of the national population concerning relevant variables like age, sex, and occupation among others; 2) the sampling frame was a true or close representation of the target population; 3) random selection was used to select the sample or a census was undertaken; 4) the likelihood of non-response bias was minimal; 5) data were collected directly from the subjects as opposed to a proxy; 6) an acceptable case definition was used in the study; 7) the study instrument used to measure the parameter of interest (condom use at the LSI) was shown to have reliability and validity; 8) the same method of data collection was used for all the participants, and 9) the numerator and denominator for condom use at the LSI were appropriate. The risk of bias in each study was summarized as follows: high when the total score was 7–9, moderate when the score was 4–6, and low when the score was 0–3.

### Data analysis

Analysis was performed in R programing language and statistical software version 3.5.2 [32]. In the primary analysis, we summarized and displayed the characteristics of the included studies in an evidence table. The primary outcome was condom use at the LSI computed as a proportion. The numerator was the number of participants that had one or more sexual partner(s) in the past 12 months (sexually active participants) and had used a condom at the LSI. The denominator was the number of sexually active participants in the primary study. In pooling the proportion of condom use at the LSI, we used the DerSimonian-Laird random-effects model, allowing for Freeman-Tukey double arcsine transformation to stabilize the variances [33].

We reported the pooled proportion of condom use at the LSI with the subsequent 95% CI and the 95% prediction interval (PI) in a forest plot. We included a 95% PI to demonstrate the variation in condom use at the LSI in different settings including the direction of evidence in future studies [34]. In the secondary outcome analysis, we applied the DerSimonian-Laird random-effects model for binary outcomes to determine the association between sex (female versus male), age (≤24 years old versus 25 years and older), and condom negotiation efficacy (high versus low) with condom use at the LSI, expressed using risk ratios (RR) with 95% CI. Similarly, we displayed the results graphically using a forest plot.

### Assessment of statistical heterogeneity

We assessed heterogeneity among the included studies using Cochran’s (Q) test and quantified using the I-squared statistics [35]. To investigate the sources of heterogeneity, we performed sub-group and random effects meta-regression analyses [36]. Here, we used the primary study characteristics, namely the study design, year of publication, country, HDI, risk of bias, and sample size.

### Publication bias assessment

We used a funnel plot and Egger’s test to assess the studies for possible publication bias. An asymmetrical funnel plot was interpreted as suggestive of publication bias [37,38]. We confirmed the asymmetry using a probability value of less than 0.1 (p<0.1) for Egger’s test [37]. We used a contour-enhanced funnel plot to aid the interpretation of funnel plot asymmetry and to differentiate between genuine publication bias and small study effect [39]. For confirmed publication bias, we applied the trim and fill analysis to estimate the number and outcome of missing studies [40].

### Sensitivity analysis

To examine the influence of the included studies on the methodological robustness, meta-analytic results, and conclusions including the study quality and sample size, we performed sensitivity analysis [41].

## Results

### Summary of study identification and screening

**Fig 1:**
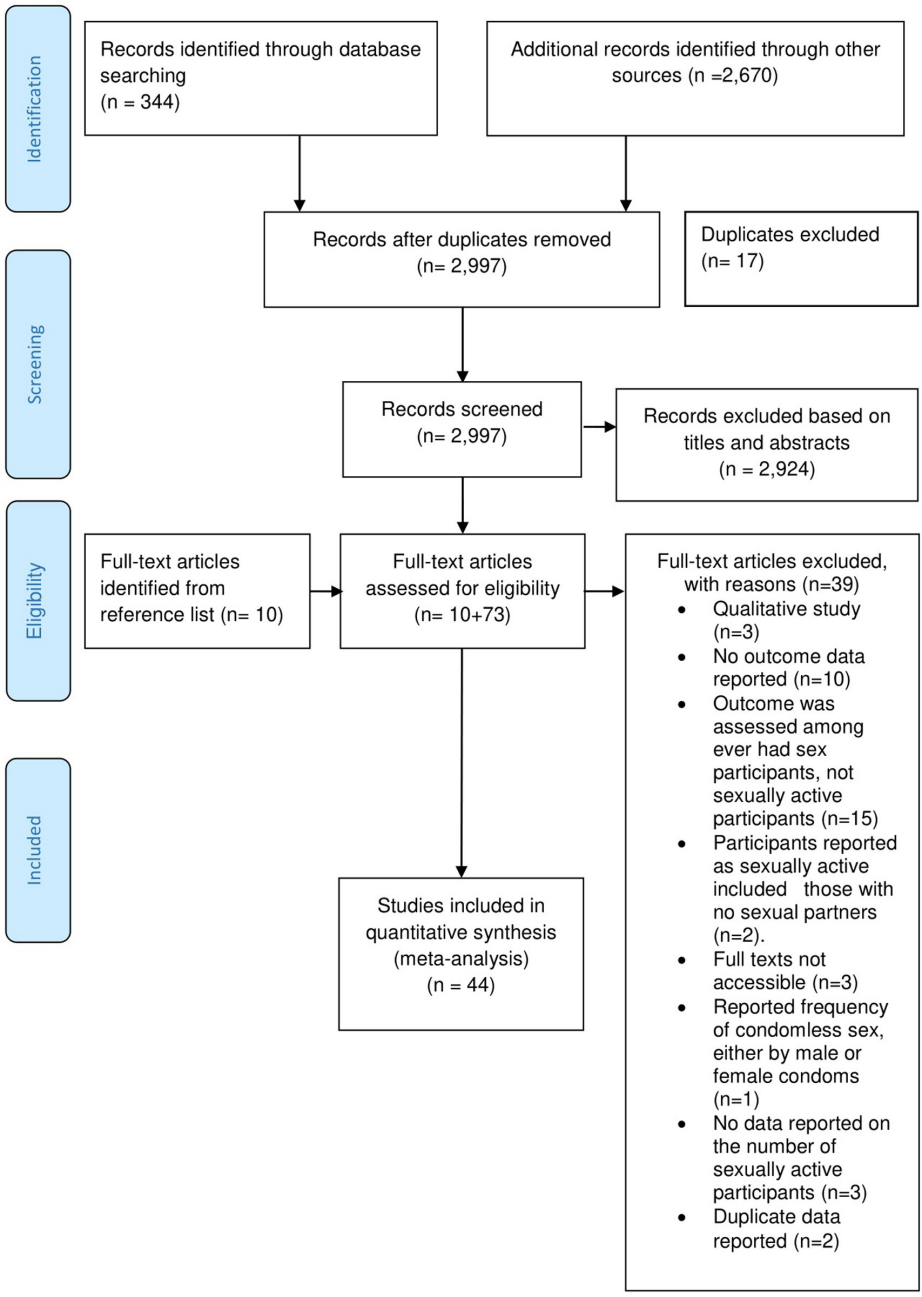
A PRISMA flow chart showing the identification, screening, and selection of primary studies.

We identified 3,014 citations: 344 through electronic databases and 2,670 through other sources. Of the identified citations, 17 duplicates were excluded leaving 2,997citations. Another 2,924 irrelevant citations were excluded after screening the titles and abstracts. Therefore, we assessed 73 full-text articles for eligibility and excluded 39 of them with reasons. From the reference list of the remaining 34 eligible full-text articles, we identified 10 additional studies. Overall (Fig 1), we meta-analyzed 44 studies [7,11–18,22–25,42–72].

### Characteristics of included studies

Table 1 presents the characteristics of the included studies. Most of the studies were from Ethiopia (n = 16) and Nigeria (n = 7). The least number of studies (n = 1) were from the Democratic Republic of Congo, Kenya, and Madagascar. Geographically, there were 23 studies from East Africa, 12 from West Africa, 8 from Southern Africa, and 1 from Central Africa. The number of studies from countries with low, medium, and HDI were 30, 10, and 4, respectively. Of the 44 studies, 40 were conducted among students at public universities, 35 involved undergraduate students, 6 included both undergraduate and postgraduate students, and 3 did not report the level of the students. Condom use at the LSI was measured in three ways: 1) *“Condom use at last sex”* in 8 studies; 2) *“Consistent condom use”* in 14 studies; and, 3) “*Always used a condom*” in 12 studies.

**Table 1:** Evidence table summarizing the characteristics of included studies.

Author and year	Country	HDI	SSA region	Sample size	Number sexually active	Frequency of condom use at LSI	Level of studies	Source of data	Risk of bias
Pelzer (2000) [42]	RSA	High	SA	206	192	73	Undergraduate	Public	Low
Fitaw and Worku (2002) [12]	Ethiopia	Low	EA	383	215	14	Not reported	Public	Moderate
Olley and Rotimi (2003) [43]	Nigeria	Low	WA	765	422	181	Mixed	Public	Moderate
Okafor and Obi (2005) [44]	Nigeria	Low	WA	950	730	182	Undergraduate	Public	Moderate
Olley (2008) [45]	Nigeria	Low	WA	583	180	96	Undergraduate	Public	Low
Rahamefy et al (2008) [11]	Madagascar	Low	EA	320	235	13	Mixed	Public	Low
Heeren et al (2009) [23]	RSA	High	SA	320	196	101	Undergraduate	Public	Low
Tagoe and Agoor (2009) [46]	Ghana	Medium	WA	334	135	106	Not reported	Public	Low
Agardh et al (2010) [22]	Uganda	Low	EA	980	480	324	Undergraduate	Public	Low
Lake Victoria Basin Commission (2010) [7]	Uganda	Low	EA	3718	2110	2050	Mixed	Both	Low
Mubita-Ngoma and Himoongna (2010) [47]	Zambia	Medium	SA	235	235	135	Undergraduate	Public	Low
Agardh et al (2011) [48]	Uganda	Low	EA	980	480	424	Undergraduate	Public	Low
Berhan et al (2011) [49]	Ethiopia	Low	EA	1220	359	208	Undergraduate	Public	Low
Fiaveh (2011) [50]	Ghana	Medium	WA	600	318	183	Mixed	Public	Low
Agardh et al (2012) [51]	Uganda	Low	EA	980	441	424	Undergraduate	Public	Low
Dingeta et al (2012) [52]	Ethiopia	Low	EA	1272	352	174	Undergraduate	Public	Low
Tura et al (2012) [15]	Ethiopia	Low	EA	1005	131	65	Undergraduate	Public	Low
Lliyasu et al (2013) [53]	Nigeria	Low	WA	375	74	36	Undergraduate	Public	Low
Masoda and Govender (2013) [54]	Democratic Republic of Congo	Low	CA	138	91	40	Undergraduate	Public	Low
Mengistu et al (2013) [55]	Ethiopia	Low	EA	390	135	75	Undergraduate	Public	Low
Nkomazana (2013) [56]	Zimbabwe	Medium	SA	345	334	180	Undergraduate	Both	Low
Akpan et al (2014) [18]	Nigeria	Low	WA	500	362	292	Undergraduate	Public	Low
Wells and Alano (2013) [57]	Ethiopia	Low	EA	916	913	608	Mixed	Public	Low
Asante et al (2014) [58]	Ghana	Medium	WA	181	93	58	Undergraduate	Public	Moderate
Ngoma et al (2014) [59]	Zambia	Medium	SA	844	331	137	Undergraduate	Public	Moderate
Negeri (2014) [60]	Ethiopia	Low	EA	860	377	209	Undergraduate	Public	Low
Sendo (2014) [61]	Ethiopia	Low	EA	207	126	22	Undergraduate	Private	Low
Shifrew et al (2014) [62]	Ethiopia	Low	EA	384	123	85	Undergraduate	Public	Low
Tobin-West et al (2014) [63]	Nigeria	Low	WA	810	589	388	Undergraduate	Public	Low
Manyumwa (2015) [64]	Zimbabwe	Medium	SA	381	232	175	Undergraduate	Public	Low
Terefe and Alemayehu (2015) [24]	Ethiopia	Low	EA	770	460	84	Undergraduate	Public	Low
Teferra et al (2015) [16]	Ethiopia	Low	EA	324	129	70	Undergraduate	Public	Low
Mavhandu-Mudsuzi (2016) [65]	Ethiopia	Low	EA	207	132	87	Undergraduate	Public	Low
Asante et al (2016) [66]	Ghana	Medium	WA	518	433	247	Undergraduate	Private	Low
Regassa et al (2016) [67]	Ethiopia	Low	EA	704	200	89	Undergraduate	Public	Low
Mamo et al (2016) [68]	Ethiopia	Low	EA	631	250	152	Undergraduate	Public	Low
Hoffman et al (2017) [69]	RSA	High	SA	576	306	196	Undergraduate	Public	Low
Akibu et al (2017) [70]	Ethiopia	Low	EA	604	328	93	Undergraduate	Public	Low
Muiga (2017) [13]	Kenya	Medium	EA	211	198	23	Undergraduate	Public	Moderate
Sakeah (2017) [71]	Ghana	Medium	WA	580	207	137	Undergraduate	Public	Low
Hafejee et al (2018) [25]	RSA	High	SA	441	287	97	Mixed	Public	Low
Muhindo et al (2018) [17]	Uganda	Low	EA	371	220	176	Undergraduate	Public	Low
Yarinbab et al (2018) [14]	Ethiopia	Low	EA	331	331	43	Not reported	Public	Low
Ajayi et al (2019) [72]	Nigeria	Low	WA	498	306	192	Undergraduate	Public	Low

Note: 1) CA: Central Africa; EA: East Africa; SA: Southern Africa; WA: West Africa; RSA: Republic of South Africa; 2) SSA: Sub-Sahara Africa; 3) HDI: Human Development Index; 4) For source of data, “Both” denotes data analyzed are from public and private universities combined, while “Public” and “Private” implies the data analyzed came from public or private universities, respectively; 5) LSI: Last sexual intercourse.

### Risk of bias and percentage agreement between the reviewers

Of the 44 studies, 38 had a low risk of bias while 6 had a moderate risk of bias (S2 Table). The percentage agreement between the two reviewers was 83.3%, which is significantly higher than the expected agreement of 62.0% (Kappa statistics = 0.46, p<0.0001).

### Condom use at the LSI: Prevalence and time trend

**Fig 2:**
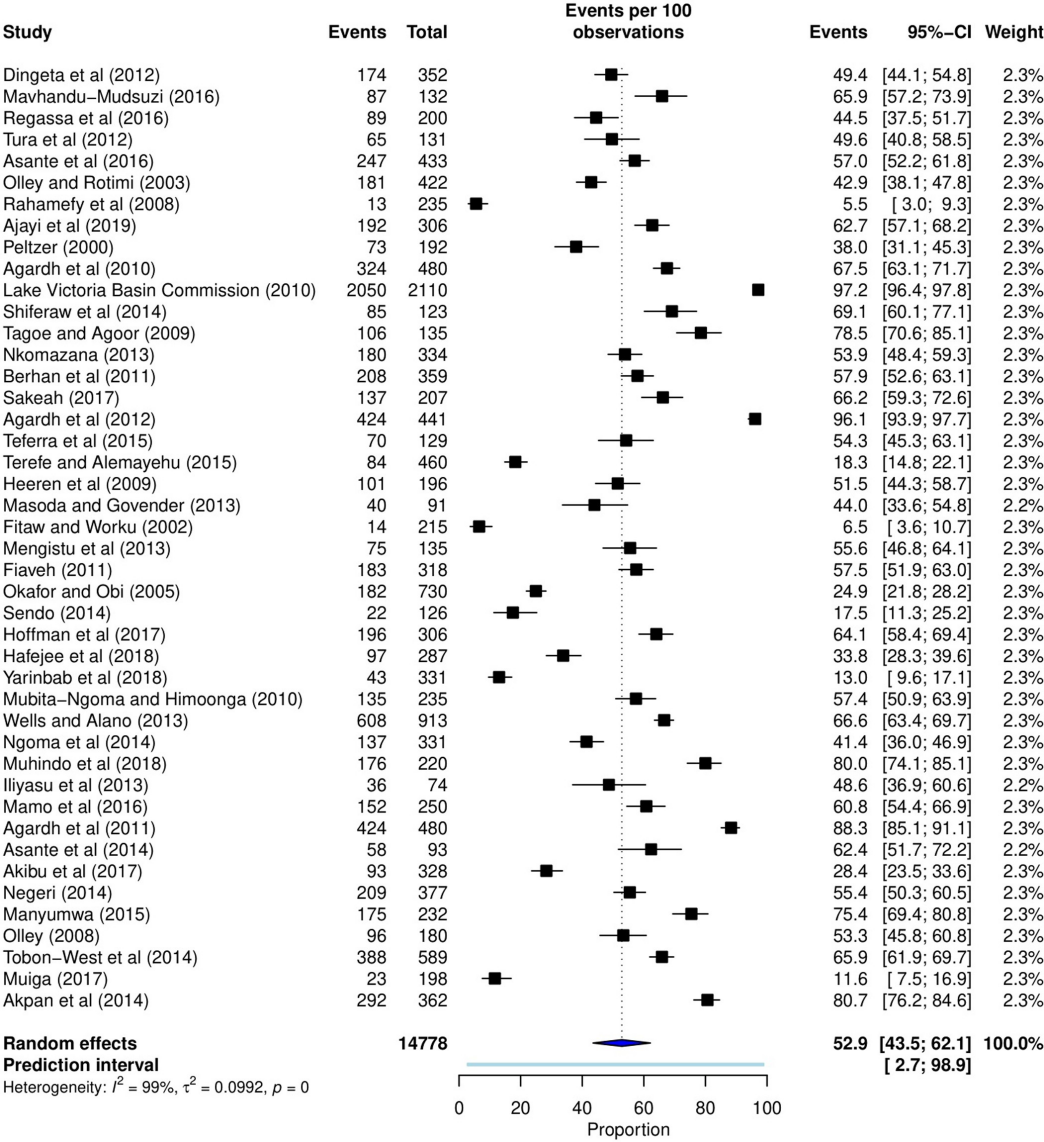
Forest plot showing the pooled proportion of condom use at the last sexual intercourse among university students in SSA.

The combined sample size for all the 44 studies [7,11–18,22–25,42–72] was 27,948 participants. Of 14,778 sexually active participants, 8,744 had used a condom at the LSI (Fig 2). Accordingly, the pooled proportion of condom use at the LSI was 52.9% (95% CI, 45.0 to 60.7%; 95% PI, 2.8 to 98.9%; I-squared = 99.0%, p< 0.0001). A time-trend analysis showed a gradual increase in condom use at the LSI between 2000 and 2019 although the increase was not statistically significant (*p* = 0.4572).

#### Sub-group analysis and sources of heterogeneity

Table 2 presents the results of the sub-group analysis. A higher proportion of condom use at the LSI was found among university students in West Africa (58.6%) than any other region in SSA: Southern Africa (50.5%), Central Africa (43.9%), and East Africa (40.5%). The proportion of condom use at the LSI using a 5-year range was as follows: 27.0% between 2000 and 2004, 40.7% between 2005 and 2009, 62.7% between 2010 and 2014, and 48.4% between 2015 and 2019. We deemed the interval sufficient to demonstrate a credible change in condom use at the LSI.

**Table 2:** Sub-group analysis and sources of heterogeneity in included studies.

Variable	Level	Number of studies	Pooled proportion of CLSI (95% CI)	I-squared value	Q-test (value, degree of freedom (df), p-value)
Sub Saharan African region	Central	1	43.9 (33.9–54.3)	-	Statistics = 4.44, df = 3, p = 0.2177
	East	23	40.5 (36.3–64.6)	99.5	
	South	8	50.5 (36.3–64.6)	95.2	
	West	12	58.6 (48.4–68.4)	97.8	
Year of publication	2000–2004	3	27.0 (0.0–84.5)	98.4	Statistics = 8.64, df = 3, P<0.034
	2005–2009	5	40.7 (8.2–78.9)	98.8	
	2010–2014	21	62.7 (52.6–72.2)	99.1	
	2015–2019	15	48.4 (35.0–61.9)	98.5	
Source of data	Public and private universities combined	2	80.5 (0.0–100.0)	99.7	Statistics = 1.70, df = 2, p = 0.426
	Private university only	2	36.2 (0.0–100.0)	98.6	
	Public university only	40	52.1 (44.2–60.1)	98.7	
Level of studies	Not reported	3	29.2 (0.0–100.0)	99.2	Statistics = 1.08, df = 2, p = 0.584
	Under and postgraduate	6	51.4 (14.8–87.2)	99.8	
	Undergraduate	35	55.2 (47.8–62.4)	98.4	
HDI	High	4	46.8 (25.6–68.6)	95.4	Statistics = 0.91, df = 2, p = 0.634
	Low	30	52.6 (41.8–63.3)	99.4	
	Medium	10	55.9 (41.1–70.1)	97.0	
Risk of bias	Moderate	6	29.4 (9.7–54.3)	97.8	Statistics = 6.77, df = 1, p = 0.01
	Low	38	56.6 (48.6–64.5)	99.2	
Measure of outcome	Always use condom	12	36.3 (23.3–50.4)	98.1	Statistics = 8.68, df = 2, p = 0.013
	Condom use at last sex	18	58.6 (47.7–69.0)	99.2	
	Consistently used condom	14	59.7 (43.3–75.2)	99.1	
Sample size	<450	20	44.7 (32.3–57.5)	98.3	Statistics = 3.66, df = 1, p = 0.056
	≥450	24	59.5 (49.7–68.9)	99.4	
Number of sexually active participants	≤350	30	47.4 (38.8–55.2)	97.9	Statistics = 3.79, df = 1, P = 0.051
	>350	14	64.1 (48.0–78.7)	99.6	

The proportion of condom use at the LSI was 80.5% for studies that combined data on students in private universities with data on students in public universities, 36.1% for studies conducted among students in private universities, and 52.1% for studies conducted among students in public universities. The proportion of condom use at the LSI was almost comparable for studies conducted among students in countries with low and medium HDI: 52.6% versus 55.6%, respectively. However, for studies conducted among students in countries with high HDI, the proportion of condom use at the LSI was 46.8%.

Concerning heterogeneity (Table 2), the year of publication (*p =* 0.034), risk of bias (*p* = 0.01), and measure of outcome (*p* = 0.013) showed statistically significant heterogeneity while the study sample size (*p* = 0.056) and number of sexually active participants (*p* = 0.051) showed borderline statistical significance.

#### Meta-regression analysis results

Table 3 shows the univariate and multivariate meta-regression analysis results. In the univariate analysis, studies published between 2010 and 2014 (Beta-coefficient (β) = 0.36, 95% CI, 0.06–0.67), studies with a lower risk of bias (β = 0.27, 95% CI, 0.06–0.49), and studies with primary outcome reported as “*condom use at the last sex*” (β = 0.22, 95% CI, 0.04–0.41) or *“consistently used condom”* (β = 0.23, 95% CI, 0.04–0.43) demonstrated statistically significant heterogeneity. In the multivariate meta-regression analysis, none of the factors was statistically significant.

**Table 3:** Univariate and multivariate meta-regression analysis.

Variable	Level	Univariate analysis	Multivariate analysis
		B-coefficient (95% CI)	B-coefficient (95% CI)
Year	2000–2004	1	1
	2005–2009	0.14 (-0.21–0.50)	0.05 (-0.31–0.40)
	2010–2014	0.36* (0.06–0.67)	0.18 (-0.13–0.50)
	2015–2019	0.22 (-0.09–0.53)	0.09 (-0.24–0.42)
Source of data	Public and private universities combined	1	
	Private university only	-0.47 (-0.98–0.04)	
	Public university only	-0.31 (-0.68–0.06)	
Level of students	Not reported	1	
	Under and postgraduate	0.23 (-0.14–0.59)	
	Undergraduate only	0.26 (-0.04–0.58)	
HDI	High	1	
	Low	0.06 (-0.22–0.34)	
	Medium	0.09 (-22-0.41)	
Quality of bias	Moderate	1	
	Low	0.27* (0.06–0.49)	0.17 (-0.07–0.40)
Measure of outcome	Always use condom	1	
	Condom use at last sex	0.22* (0.04–0.41)	0.14 (-0.05–0.32)
	Consistently used condom	0.23* (0.04–0.43)	0.14 (-0.06–0.33)
Sample size	<450	1	
	≥450	0.15 (-0.006–0.301)	
Number of sexually active participants	≤350	1	1
	>350	0.17* (0.01–0.33)	0.12 (-0.04–0.28)

Note: Statistical significance codes: *** p<0.001;

** p<0.01;

* p< 0.05.

#### Publication bias and small study effect findings

We found an asymmetrical distribution of studies in a funnel plot suggesting possible publication bias. However, in a contour-enhance funnel plot, smaller studies were distributed in the regions of both statistical and non-statistical significance, suggesting that the asymmetry might have resulted from several factors but not solely publication bias. Egger’s test was statistically significant, with the 95% CI of the intercept excluding zero (Bias (intercept), -6.09; 95% CI, -9.90, -2.28; p = 0.002). This meant that the asymmetry resulted from a small study effect. Accordingly, trim and fill analysis was not performed.

#### Sensitivity analysis findings

The exclusion of one study at a time generated a new pooled proportion of condom use at the LSI that fell within the 95% CI of the original pooled result. This meant that the meta-analytic results and conclusions are robust to methodological quality, meta-analytic approach, study quality, and the sample size.

### Association between sex, age, and condom negotiation efficacy with condom use at the last sexual intercourse (LSI)

**Fig 3:**
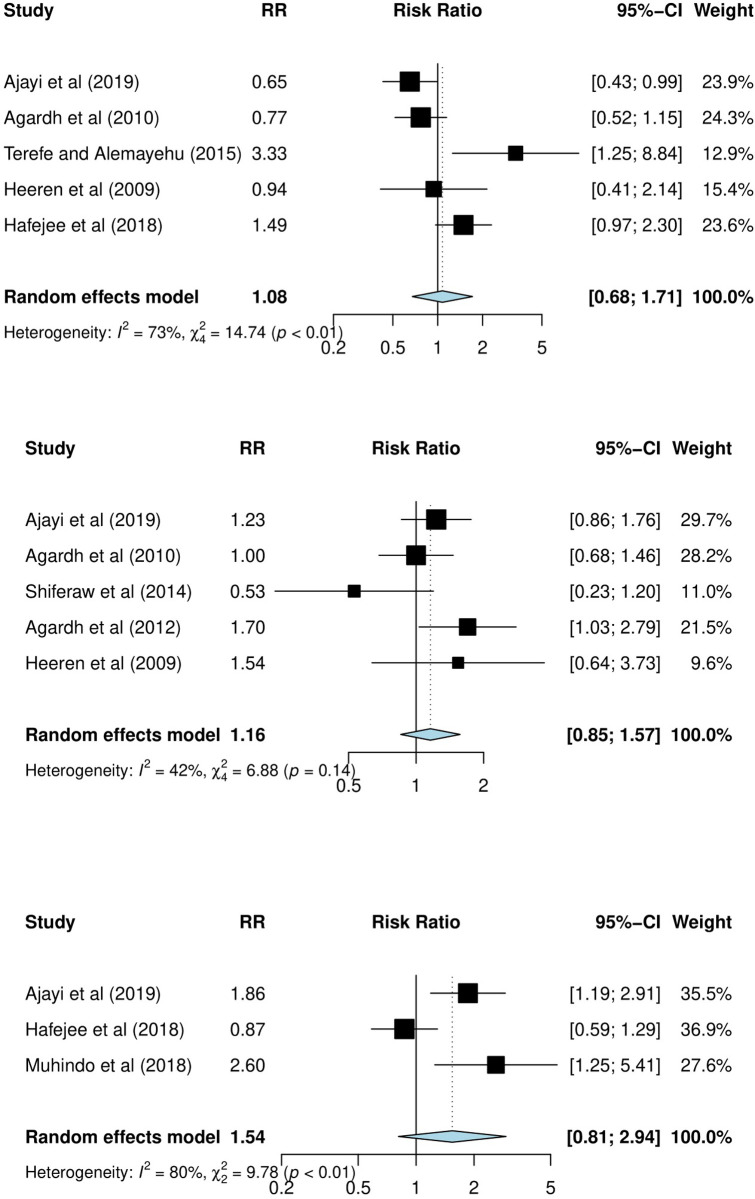
Panel of three forest plots showing the pooled association between sex, age, and condom negotiation efficacy with condom use at the LSI among university students in SSA. Fig 3A shows condom use at the LSI among female versus male students. Fig 3B shows condom use at the LSI among younger (≤24 years old) versus older students (25 years and more). Fig 3C shows the condom use at LSI among students with a high versus low condom negotiation.

Fig 3 summarizes the association between sex (5 studies), age (5 studies), and condom negotiation efficacy (3 studies) with condom use at the LSI. Fig 3A shows that condom use at the LSI was more likely among female than male students (pooled RR = 1.08; 95% CI, 0.68–1.71; I-squared = 73%, p<0.01). Fig 3B shows that condom use at the LSI was more likely among younger students (≤24 years old) compared to students aged 25 years and more (pooled RR, 1.16; 95% CI, 0-85-1.57; I-squared = 42%, p = 0.14).

Fig 3C indicates that students with a higher condom negotiation efficacy were more likely to use a condom at the LSI compared to students with a lower condom negotiation efficacy (pooled RR, 1.54; 95% CI, 0-81-2.94; I-squared = 80.0%, p<0.01).

## Discussion

We studied condom use at the LSI among university students in SSA. Condom at the LSI measures progress towards preventing exposure to HIV through condomless sexual intercourse among people at a higher risk of STIs, such as university students. Improving access to condoms through condom availability programs (defined as interventions at school, city, state, or federal levels that provide improved access to condoms) is important in achieving better sexual health outcomes among university students. Previous studies have reported that condom availability programs that target students lower the age at sexual initiation, increase condom use at the LSI [73], and reduce the risk of STI acquisition [74] including the prevalence of STI symptoms [75,76], and importantly, by no means increase sexual activity and multiple sexual partnerships. We found that merely 52.9% of university students in SSA had used a condom at the LSI. The study further found a tendency towards higher condom use at the LSI among female than male students, younger than older students, and students with a higher condom negotiation efficacy compared to those with a lower condom negotiation efficacy.

Our data also show that condom use at the LSI among university students in SSA increased between 2000 and 2019. The pooled proportion of condom use at the LSI in this study was remarkably lower than the 80–90% condom use at the LSI observed across most countries globally [77]. Although several reasons might plausibly explain the low use of a condom at the LSI, we present a few of them. First, across most countries in SSA, young people have poor access to condoms and face stigma including age restrictions, and religious and gender barriers to accessing condoms. Second, some countries have laws that prohibit young people from carrying condoms and people from promoting and distributing condoms at venues where young people usually socialize. Third, besides the fall in international funding for condom procurement, most countries in SSA have limited or no domestic funding for condom procurement leading to a scarcity of condoms. For instance, in 2015, six billion condoms were needed in SSA but only 2.7 billion were distributed, representing at least a 50% shortfall [77]. The low condom use at the LSI has important consequences among sexually active university students as it results in STI acquisition including HIV [78,79] and unwanted pregnancies.

Our study found that sex, age, and condom negotiation efficacy were not associated with condom use at the LSI, implying that HIV prevention strategies such as risk reduction counseling and condom campaigns should target all sexually active university students to reduce the risk of acquisition of STIs including HIV [80]. Our findings also underscore the importance of improving access to HIV pre [81] and post-exposure prophylaxis to prevent HIV acquisition among university students in SSA. The findings further emphasize the importance of access to emergency contraception among university students in SSA to prevent unwanted pregnancies. Additionally, there is a need to tackle the barriers to and inequities in condom access and use among university students in SSA.

We found increased use of a condom at the LSI between 2000 and 2019, indicating that condom promotion campaigns are achieving the desired effect of reaching individuals who engage in high-risk sexual relationships [82]. Therefore, universities in SSA need to sustain the increase in condom use at the LSI.

### Study strengths and limitations

Our study has some important strengths. To the best of our understanding, this is the first study to highlight condom use at the LSI among university students in SSA. The data analyzed were from almost all the regions in SSA. We meta-analyzed data for sexually active students in pooling the proportion of condom use at the LSI, making our results less biased. Our findings are robust to the methodological quality and analytic approach since no study had a significant influence on the overall meta-analytic results and conclusions. The risk of bias was low in the majority of the studies. Nonetheless, there are limitations to consider. All the 44 studies meta-analyzed employed a cross-sectional design which is characterized by limitations of selection bias and confounding. However, this limitation was mitigated by abstracting data for only adjusted measures of effect. Also, there is a likelihood that the outcome measures *“Consistent condom use*” and *“Always used condom”* might have been undermined by recall and reporting biases. For instance, male students might perceive condom use at the LSI as suggestive of low masculinity. Relatedly, in settings where condom promotion campaigns exist, there is a possibility that male students are more likely to report condom use at the LSI even when this was not the case. Nonetheless, both measures of outcome signify condom use at the LSI. The exclusion of studies published in non-English languages such as French, Portuguese, and Arabic among others might have led to an inaccurate measure of condom use at the LSI.

The studies meta-analyzed were statistically heterogeneous despite the use of an appropriate analytic approach (random-effects model) to pool the data and additional measures (sub-group and meta-regression analyses) taken to investigate and explain the sources of statistical heterogeneity. We emphasize a need to cautiously interpret the results as the analytic approaches do not eliminate statistical heterogeneity. Also, several factors that contribute to heterogeneity such as time since LSI, preventive measures other than a condom, type of sexual activity, urban versus rural residence, power relationship with a sexual partner, covariates used for statistical adjustments, and methods used for collecting information on condom use among others, were not measured.

## Conclusions and recommendations

Our study found a 52.9% prevalence of condom use at the LSI among sexually active university students in SSA, which is considerably lower than expected. The low proportion of condom use at the LSI places these predominantly young adults at a higher risk of acquisition of STIs including HIV. With regards to sex, age, and condom negotiation efficacy, we found similar use of a condom at the LSI. Therefore, there is a need for context-relevant campaigns to advocate for use of condoms among university students in SSA.

## Supporting information

S1 Table PRISMA checklist. (DOC)

S2 Table Detailed summary of the risk of bias findings. (DOCX)

S1 File Search strategy in PubMed. (DOCX)
