# Supplementary material for: Low condom use at the last sexual intercourse among university students in sub-Saharan Africa: Evidence from a systematic review and meta-analysis
Source: PLoS One. 2022 Aug 10;17(8):e0272692. doi: 10.1371/journal.pone.0272692 (PMC9365151; doi:10.1371/journal.pone.0272692)
Supplement: S2 Table — (DOCX) [file pone.0272692.s002.docx]

**S1 Table 2: Detailed summary of the risk of bias findings**

| **Author (year)** | **Country** | **Nine items assessed** | | | | | | | | | **Total** | **Risk of bias** |
| --- | --- | --- | --- | --- | --- | --- | --- | --- | --- | --- | --- | --- |
|  |  | **1** | **2** | **3** | **4** | **5** | **6** | **7** | **8** | **9** |  |  |
| Pelzer (2000) [[42](#_ENREF_42)] | Republic of South Africa | 1 | 0 | 0 | 0 | 0 | 1 | 0 | 0 | 1 | 3 | Low |
| Fitaw and Worku (2002) [[12](#_ENREF_12)] | Ethiopia | 0 | 1 | 0 | 1 | 0 | 0 | 1 | 0 | 0 | 3 | Moderate |
| Olley and Rotimi (2003) [[43](#_ENREF_43)] | Nigeria | 0 | 1 | 1 | 1 | 0 | 0 | 1 | 0 | 1 | 5 | Moderate |
| Okafor and Obi (2005) [[44](#_ENREF_44)] | Nigeria | 1 | 0 | 0 | 0 | 0 | 1 | 1 | 0 | 1 | 4 | Moderate |
| Olley (2008) [[45](#_ENREF_45)] | Madagascar | 0 | 0 | 0 | 0 | 0 | 0 | 0 | 0 | 0 | 0 | Low |
| Rahamefy et al (2008) [[11](#_ENREF_11)] | Nigeria | 1 | 0 | 1 | 1 | 0 | 0 | 0 | 0 | 0 | 3 | Low |
| Heeren et al (2009) [[23](#_ENREF_23)] | Ghana | 0 | 0 | 0 | 1 | 0 | 1 | 0 | 0 | 1 | 3 | Low |
| Tagoe and Agoor (2009) [[46](#_ENREF_46)] | Republic of South Africa | 0 | 1 | 1 | 0 | 0 | 0 | 1 | 0 | 0 | 3 | Low |
| Agardh et al (2010) [[22](#_ENREF_22)] | Uganda | 0 | 0 | 0 | 0 | 0 | 0 | 1 | 0 | 0 | 1 | Low |
| Lake Victoria Basin Commission (2010) [[7](#_ENREF_7)] | Zambia | 0 | 0 | 0 | 0 | 0 | 1 | 0 | 0 | 1 | 2 | Low |
| Mubita-Ngoma and Himoongna (2010) [[47](#_ENREF_47)] | Uganda | 0 | 0 | 1 | 0 | 0 | 0 | 1 | 0 | 0 | 2 | Low |
| Agardh et al (2011) [[48](#_ENREF_48)] | Uganda | 0 | 0 | 1 | 0 | 0 | 0 | 1 | 0 | 0 | 2 | Low |
| Berhan et al (2011) [[49](#_ENREF_49)] | Ethiopia | 0 | 0 | 0 | 0 | 0 | 0 | 1 | 0 | 0 | 1 | Low |
| Fiaveh (2011) [[50](#_ENREF_50)] | Ghana | 0 | 0 | 0 | 0 | 0 | 0 | 1 | 0 | 0 | 1 | Low |
| Agardh et al (2012) [[51](#_ENREF_51)] | Ethiopia | 0 | 0 | 0 | 0 | 0 | 1 | 0 | 0 | 1 | 2 | Low |
| Dingeta et al (2012) [[52](#_ENREF_52)] | Uganda | 0 | 0 | 1 | 0 | 0 | 0 | 1 | 0 | 0 | 2 | Low |
| Tura et al (2012) [[15](#_ENREF_15)] | Ethiopia | 0 | 0 | 0 | 0 | 0 | 0 | 1 | 0 | 0 | 1 | Low |
| Lliyasu et al (2013) [[53](#_ENREF_53)] | Ethiopia | 0 | 0 | 0 | 0 | 0 | 0 | 0 | 0 | 1 | 1 | Low |
| Masoda and Govender (2013) [[54](#_ENREF_54)] | Ethiopia | 0 | 0 | 0 | 1 | 0 | 0 | 1 | 0 | 0 | 2 | Low |
| Mengistu et al (2013) [[55](#_ENREF_55)] | Nigeria | 0 | 0 | 0 | 0 | 0 | 1 | 0 | 0 | 1 | 2 | Low |
| Nkomazana (2013) [[56](#_ENREF_56)] | Zimbabwe | 0 | 1 | 0 | 1 | 0 | 0 | 1 | 0 | 0 | 3 | Low |
| Akpan et al (2014) [[18](#_ENREF_18)] | Democratic Republic of Congo | 0 | 0 | 0 | 0 | 0 | 0 | 0 | 0 | 0 | 0 | Low |
| Wells and Alano (2013) [[57](#_ENREF_57)] | Ghana | 0 | 1 | 1 | 1 | 0 | 1 | 1 | 0 | 0 | 5 | Moderate |
| Asante et al (2014) [[58](#_ENREF_58)] | Ethiopia | 0 | 0 | 0 | 0 | 0 | 0 | 0 | 0 | 0 | 0 | Low |
| Ngoma et al (2014) [[59](#_ENREF_59)] | Nigeria | 0 | 0 | 0 | 0 | 0 | 0 | 0 | 0 | 0 | 0 | Low |
| Negeri (2014) [[60](#_ENREF_60)] | Nigeria | 0 | 0 | 0 | 0 | 0 | 0 | 1 | 0 | 0 | 1 | Low |
| Sendo (2014) [[61](#_ENREF_61)] | Zambia | 0 | 0 | 0 | 1 | 0 | 1 | 1 | 0 | 1 | 4 | Moderate |
| Shifrew et al (2014) [[62](#_ENREF_62)] | Ethiopia | 0 | 0 | 0 | 0 | 0 | 0 | 0 | 0 | 0 | 0 | Low |
| Tobin-West et al (2014) [[63](#_ENREF_63)] | Ethiopia | 0 | 0 | 0 | 0 | 0 | 0 | 1 | 0 | 1 | 2 | Low |
| Manyumwa (2015) [[64](#_ENREF_64)] | Ethiopia | 0 | 0 | 0 | 0 | 0 | 0 | 1 | 0 | 1 | 2 | Low |
| Terefe and Alemayehu (2015) [[24](#_ENREF_24)] | Ethiopia | 0 | 0 | 0 | 0 | 0 | 0 | 1 | 0 | 0 | 1 | Low |
| Teferra et al (2015) [[16](#_ENREF_16)] | Zimbabwe | 0 | 0 | 0 | 0 | 0 | 0 | 0 | 0 | 0 | 0 | Low |
| Mavhandu-Mudsuzi (2016) [[65](#_ENREF_65)] | Ethiopia | 0 | 0 | 0 | 0 | 0 | 0 | 0 | 0 | 0 | 0 | Low |
| Asante et al (2016) [[66](#_ENREF_66)] | Ethiopia | 0 | 0 | 0 | 0 | 0 | 0 | 0 | 0 | 0 | 0 | Low |
| Regassa et al (2016) [[67](#_ENREF_67)] | Ethiopia | 0 | 0 | 0 | 0 | 0 | 1 | 0 | 0 | 1 | 2 | Low |
| Mamo et al (2016) [[68](#_ENREF_68)] | Ghana | 0 | 0 | 0 | 0 | 0 | 0 | 1 | 0 | 0 | 1 | Low |
| Hoffman et al (2017) [[69](#_ENREF_69)] | Kenya | 1 | 1 | 1 | 0 | 0 | 1 | 0 | 0 | 0 | 4 | Moderate |
| Akibu et al (2017) [[70](#_ENREF_70)] | Ethiopia | 0 | 0 | 0 | 0 | 0 | 0 | 0 | 0 | 0 | 0 | Low |
| Muiga (2017) [[13](#_ENREF_13)] | Ghana | 0 | 0 | 0 | 0 | 0 | 0 | 0 | 0 | 0 | 0 | Low |
| Sakeah (2017) [[71](#_ENREF_71)] | Republic of South Africa | 0 | 0 | 0 | 0 | 0 | 0 | 0 | 0 | 0 | 0 | Low |
| Hafejee et al (2018) [[25](#_ENREF_25)] | Republic of South Africa | 0 | 0 | 0 | 0 | 0 | 0 | 0 | 0 | 0 | 0 | Low |
| Muhindo et al (2018) [[17](#_ENREF_17)] | Ethiopia | 0 | 0 | 0 | 0 | 0 | 0 | 0 | 0 | 0 | 0 | Low |
| Yarinbab et al (2018) [[14](#_ENREF_14)] | Uganda | 1 | 1 | 0 | 0 | 0 | 0 | 0 | 0 | 0 | 2 | Low |
| Ajayi et al (2019) [[72](#_ENREF_72)] | Nigeria | 0 | 0 | 0 | 0 | 0 | 0 | 1 | 0 | 0 | 1 | Low |

**Note items assessed**: 1) the study’s target population was a close representation of the national population concerning relevant variables like age, sex, and occupation among others; 2) the sampling frame was a true or close representation of the target population; 3) random selection was used to select the sample or a census was undertaken; 4) the likelihood of non-response bias was minimal; 5) data were collected directly from the subjects as opposed to a proxy; 6) an acceptable case definition was used in the study; 7) the study instrument used to measure the parameter of interest (condom use at the LSI) was shown to have reliability and validity; 8) the same method of data collection was used for all the participants, and 9) the numerator and denominator for condom use at the LSI were appropriate.
