# Supplementary material for: Low condom use at the last sexual intercourse among university students in sub-Saharan Africa: Evidence from a systematic review and meta-analysis
Source: PLoS One. 2022 Aug 10;17(8):e0272692. doi: 10.1371/journal.pone.0272692 (PMC9365151; doi:10.1371/journal.pone.0272692)
Supplement: S1 File — (DOCX) [file pone.0272692.s003.docx]

**S1 File 1: Search strategy in PubMed**

("University student"[All Fields] OR "University students"[All Fields]) AND ((("condom s"[All Fields] OR "condoms"[MeSH Terms] OR "condoms"[All Fields] OR "condom"[All Fields]) AND "last"[All Fields] AND ("sex"[MeSH Terms] OR "sex"[All Fields])) OR (("condom s"[All Fields] OR "condoms"[MeSH Terms] OR "condoms"[All Fields] OR "condom"[All Fields]) AND "last"[All Fields] AND ("coitus"[MeSH Terms] OR "coitus"[All Fields] OR ("sexual"[All Fields] AND "intercourse"[All Fields]) OR "sexual intercourse"[All Fields])) OR (("condom s"[All Fields] OR "condoms"[MeSH Terms] OR "condoms"[All Fields] OR "condom"[All Fields]) AND "latest"[All Fields] AND ("occasion"[All Fields] OR "occasions"[All Fields])) OR (("condom s"[All Fields] OR "condoms"[MeSH Terms] OR "condoms"[All Fields] OR "condom"[All Fields]) AND ("recent"[All Fields] OR "recently"[All Fields] OR "recents"[All Fields]) AND ("sex"[MeSH Terms] OR "sex"[All Fields])) OR "consistent condom use"[All Fields] OR (("consistence"[All Fields] OR "consistences"[All Fields] OR "consistencies"[All Fields] OR "consistency"[All Fields] OR "consistent"[All Fields] OR "consistently"[All Fields]) AND ("condom s"[All Fields] OR "condoms"[MeSH Terms] OR "condoms"[All Fields] OR "condom"[All Fields])) OR (("inconsistencies"[All Fields] OR "inconsistency"[All Fields] OR "inconsistent"[All Fields] OR "inconsistently"[All Fields]) AND ("condom s"[All Fields] OR "condoms"[MeSH Terms] OR "condoms"[All Fields] OR "condom"[All Fields])) OR "Inconsistent condom use"[All Fields] OR ("condom s"[All Fields] OR "condoms"[MeSH Terms] OR "condoms"[All Fields] OR "condom"[All Fields]))
